# A Knowledge, Attitudes and Practices Survey Conducted Three Years after Halting Ivermectin Mass Treatment for Onchocerciasis in Guatemala

**DOI:** 10.1371/journal.pntd.0004777

**Published:** 2016-06-24

**Authors:** Frank O. Richards, Robert E. Klein, Oscar de León, Renata Mendizábal-Cabrera, Alba Lucía Morales, Vitaliano Cama, Carol G. Crovella, Carlos E. Díaz Espinoza, Zoraida Morales, Mauricio Sauerbrey, Nidia Rizzo

**Affiliations:** 1 River Blindness Elimination Program, Lymphatic Filariasis Elimination Program, and Schistosomiasis Control Program, The Carter Center, Atlanta, Georgia, United States of America; 2 Center for Health Studies (CHS), Universidad del Valle de Guatemala (UVG), Guatemala City, Guatemala; 3 Onchocerciasis Elimination Program for the Americas, The Carter Center, Guatemala City, Guatemala; 4 Division of Parasitic Diseases and Malaria, Centers for Disease Control and Prevention, Atlanta, Georgia, United States of America; 5 Onchocerciasis Sub-Program, National Ministry of Health, Guatemala City, Guatemala; Emory University, UNITED STATES

## Abstract

**Background:**

Mass drug administration (MDA) with ivermectin for onchocerciasis was provided in Guatemala’s Central Endemic Zone (CEZ) over a 24 year period (1988–2011). Elimination of *Onchocerca volvulus* transmission was declared in 2015 after a three year post MDA surveillance period (2012–2014) showed no evidence of recrudescence. The purpose of the present study was to evaluate the knowledge, attitudes and practices (KAP) towards onchocerciasis and ivermectin among residents in the post endemic CEZ. A major interest in this study was to determine what community residents thought about the end of the ivermectin MDA program.

**Methodology/Principal Findings:**

A total of 148 interviews were conducted in November 2014 in four formerly hyperendemic communities using a standard questionnaire on smart phones. The majority (69%) of respondents knew that the MDA program had ended because the disease was no longer present in their communities, but a slight majority (53%) was personally unsure that onchocerciasis had really been eliminated. Sixty-three percent wanted to continue to receive ivermectin because of this uncertainty, or because ivermectin is effective against intestinal worms. Eighty-nine percent of respondents said that they would seek medical attention immediately if a family member had symptoms of onchocerciasis (especially the presence of a nodule), which is a finding very important for ongoing surveillance.

**Conclusions/Significance:**

Many respondents wanted to continue receive ivermectin and more than half did not believe onchocerciasis had been eliminated. The ministry of health outreach services should be prepared to address ongoing concerns about onchocerciasis in the post endemic CEZ.

## Author Summary

Human onchocerciasis (also known as ‘River Blindness’) is a parasitic disease of the skin and eyes whose etiological agent (*Onchocerca volvulus*) is transmitted by black flies. Guatemala was the country in the Americas most afflicted by onchocerciasis, and the largest focus of onchocerciasis in Guatemala was the Central Endemic Zone (CEZ). In the late 1980’s the Guatemalan ministry of health launched an onchocerciasis transmission elimination program based on mass drug administration (MDA) with the medicine ivermectin (Mectizan) in affected communities. Health education messages based on community knowledge, attitudes and practices (KAP) surveys were designed to enhance understanding and sustain participation in the MDA program. The first KAP survey (published 1991) recommended the elements of the health education program. The second KAP survey (published 1995) identified barriers preventing the achievement of adequate treatment coverage, and recommended adjustments to program activities to improve participation. This third KAP survey was conducted in 2014, almost 3 years after cessation of the MDA program. Results indicated that many respondents wanted ivermectin MDA to continue in their community because of uncertainty about onchocerciasis elimination, or in order to treat intestinal worms. The ministry of health outreach services should be prepared to address ongoing concerns of the populations residing in post endemic, post MDA onchocerciasis Guatemala.

## Introduction

Human onchocerciasis (River Blindness) is caused by the filarial nematode *Onchocerca volvulus* that is transmitted by the bite of infected *Simulium* black flies that breed in rapid flowing rivers and streams. The adult worms of *O*. *volvulus* live mainly in subcutaneous nodules, which are often palpable, where the fertilized female worms during their 9–14 years of reproductive life release embryos called microfilariae. The microfilariae are predominantly found in the dermis where they can be ingested by female black fly during a blood meal. Microfilariae so ingested can develop into infective third stage larvae, which may then be transmitted to another person. No animal reservoirs exist. The great majority of microfilariae eventually die in the human tissues, causing complex immunological mediated manifestations leading to skin disease, lymphatic complications and ocular lesions. [1,2]

Onchocerciasis occurs primarily in Africa, but Yemen and six countries in the Americas are also affected [3,4] although the transmission of the parasite has been eliminated in Colombia [5], Ecuador [6], Mexico [7], and (it is believed) Guatemala [8]. In Guatemala, onchocerciasis transmission occurred in four geographically distinct transmission zones (‘foci’) and about half of the 220,000 persons at risk in the country resided in the focus known as the Central Endemic Zone (CEZ) [8,9]. The Ministry of Public Health and Social Assistance (MSPAS), began onchocerciasis control efforts in 1935 with the surgical removal of nodules by uniformed health workers organized into brigades [9,10]. The decision by Merck & Company in 1987 to donate ivermectin (Mectizan) resulted in a shift to a strategy from nodulectomy to mass drug administration (MDA) that became operational in all Guatemalan foci by the year 2000 [8].

There were important challenges to establishing an ivermectin based MDA strategy. First, a single dose of ivermectin does not kill the adult parasites, only the microfilariae. A single dose of ivermectin prevents repopulation of the skin with microfilaria for about six months [1]. To block transmission of the parasite by the vectors, ivermectin needs to be given twice per year with high treatment coverage (>85% of the MDA eligible population, which excludes children under five years of age and pregnant or mothers breastfeeding infants younger than one month old [1]. Second, high treatment coverage needs to be maintained for many years until the adult worm population in the transmission zone becomes incapable of sustaining itself, even if MDA is stopped [11]. Third, initial ivermectin treatment is often associated with localized or systemic allergic reactions, and fever, resulting from the destruction of microfilaria. While these reactions diminish with repeated treatments, fear of getting sick from the medicine was an important cause of low MDA uptake (coverage) early in the Guatemalan MDA program [12,13]. Fourth (and what can be seen as an advantage perhaps rather than a challenge) ivermectin is also effective against most ectoparasites (lice, scabies) and many intestinal helminthes (especially the human roundworm, *Ascaris lumbricoides*) [14,15]. The visible and valued experience of passing these large worms commonly led populations under MDA to value the deworming effect associated with the treatment above that for which the health education messages (onchocerciasis) have been designed [13,16].

In order to understand community values and concerns related to onchocerciasis and ivermectin MDA, two knowledge attitude and practices (KAP) surveys were conducted in the early years of the onchocerciasis program by our group. The results from the surveys were to be used to design health education messages and communication strategies to improve levels of participation in the MDA program. The first survey was conducted in communities that had not yet experienced ivermectin. It indicated that people used the term ‘*filaria*’ to describe onchocerciasis, which was defined as a nodule under the skin that could somehow affect the vision; surgical removal of the nodule (nodulectomy) by MSPAS brigades was the only recognized treatment. Transmission of *filaria* by a biting insect was largely understood, but the term microfilaria (the target of the ivermectin) was not recognized. In a free listing of diseases afflicting the communities, followed by their ranking based on paired comparisons, we learned that respondents ranked *filaria* 13^th^ in seriousness compared to other commonly recognized conditions afflicting their communities [16].

Our second KAP survey was prompted by the observation that residents in some communities refused to take ivermectin when offered [13]. This survey was focused on exploring community concerns and the reasons for low participation rates. Fear of adverse reactions following treatment was the principal reason for rejecting treatment. Health education messages to address these concerns were designed to inform the community more about the complex lifecycle of the parasite, and explain that as the numbers of microfilaria diminished in the body with frequent treatment, the severity of adverse reactions would diminish.

A cumulative total of 2.9 million ivermectin treatments were delivered in the 321 communities targeted by the CEZ MDA program between 1988 and 2011. The biannual (every six months) treatments were delivered as directly observed treatments at the community level, and after the year 2000, treatment coverage exceeded 85% of the eligible population. Based on the epidemiological assessments that showed no infection in residents and vectors sampled in sentinel areas, and very low (<0.1%) *O*. *volvulus* antibody in young children in a population-based survey throughout the CEZ, MDA was halted in 2012. A three-year post treatment surveillance period (PTS) was launched during which health workers continued to visit the communities to explain the reason for discontinuation of MDA and to promote the need for continued vigilance to ensure disease elimination and monitoring for a possible recrudescence of the disease. PTS ended in 2014 with the successful completion of an entomological survey in sentinel and non-sentinel areas that showed continued interruption of transmission [8].

The KAP study reported herein was conducted in November 2014, thirty four months after MDA had ended. It explored community resident’s understanding of the reason for ending the MDA program and the depth of the belief of its success. It also included a reexamination of their knowledge of onchocerciasis, and its seriousness compared to other diseases.

## Materials and Methods

### Study villages

**Fig 1:**
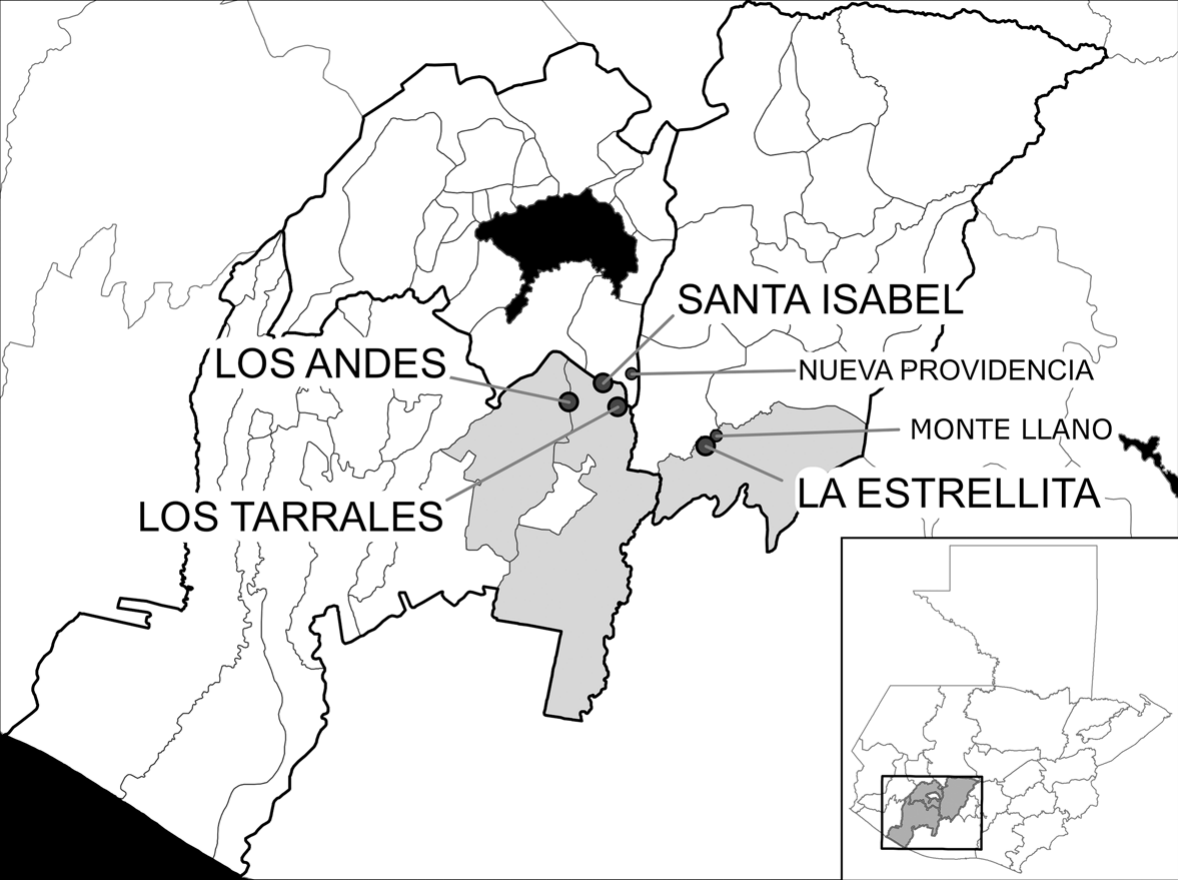
Names and locations of study communities included in the KAP survey in the Central Endemic Zone (CEZ) for onchocerciasis, Guatemala. The questionnaires were pre-tested in Nueva Providencia and Monte Llano (shown using a smaller font size). The grey color in the inset outlines the departments in which the CEZ is located. The grey color in the main map shows the counties (*municipios*) where the four study communities were located. The black color represents Lake Atítlan.

The study was conducted in 2014 in three privately-owned coffee plantations (“*fincas*”) in the Department of Suchitepéquez [Santa Isabel (14°32'44", -91°9'14"), Los Tarrales (14°31'18",-91°8'14"), and Los Andes (14°31'37",-91°11'25")] (Fig 1) previously surveyed in the second (1992) KAP survey [13]. A fourth 2014 study site was an independent community [La Estrellita (14°28'51",-91°2'52")] in the Department of Chimaltenango that had not been previously surveyed in KAP studies. All four communities were within a radius of 15 km of survey sites in the first KAP study. In addition, all four communities were sentinel villages for the onchocerciasis program and as such had more frequent interactions with the Ministry of Health teams through the years [8]. Prior to the MDA intervention, prevalence of microfilaria in skin biopsies in the surveyed villages were: Los Andes 74% (based on a survey in 1988), Santa Isabel 90% (1981), Los Tarrales 65% (1981) and La Estrellita 56% (1994). Surveys in these villages conducted in 2007–2010 showed no microfilaria infection (prevalence 0%) [8,17].

### Sample

A stratified random sampling by location was originally devised to estimate proportions of variables with binomial distribution [18], applying the finite population correction, considering an α = 0.05, a ratio of 0.5, a 90% of response rate and a design effect of 1.5 to account for the decrease of precision due to clustering. Due to the low number of households in each community, and considering the sample size increase after adjusting for response rate and the design effect, it was decided to include all occupied households (census) in the survey to ensure the highest possible precision in the estimates for each community.

Male and female heads of household with a minimum of six years of community residence were interviewed. When the head-of-household or an adult family member was not present, a return visit was scheduled for the next day, and if still absent, a third visit was scheduled. If, after three visits to the same household, the head–of-household or a responsible adult resident of the household was not present, the household was not included in the survey.

### Interviews

The questionnaires were designed to take approximately 30 minutes and included open and closed questions organized into five sections: (1) All illnesses affecting community residents, (2) onchocerciasis (*filaria*) symptoms and transmission, (3) the treatment of onchocerciasis, (4) the reasons for halting MDA, and 5) the post-treatment period. Surveys were conducted by field technicians from the Universidad Del Valle de Guatemala and responses of participants were recorded on smartphones. The questionnaires and smartphone programs were pre-tested in the CEZ communities Nueva Providencia and Monte Llano located in the Departments of Sololá and Chimaltenango, respectively (Fig 1).

To explore the perceived seriousness of onchocerciasis a list of Illnesses was created by the respondents by using the same methodology applied in the original KAP survey, asking the questions “What illnesses affect this community?” “What else?” “What else?” The 15 most frequently reported illnesses were selected and paired with every other illness to generate 105 pairs. Subsequently, using a paired comparison format, the 105 illness pairs were randomized and presented orally to a subsample of heads of households in the four communities. Each person was asked to indicate the illness in each pair that they considered to be the most serious (*grave*). This resulted in a list of the 15 most common illnesses ranked in order of perceived seriousness. The ranking of perceived seriousness of common illnesses generated in the 2014 survey was compared with the ranking generated by respondents in our 1991 survey.

### Analysis

The unit of analysis for the survey was the individual respondent. The answers to open-ended questions were coded and organized in major categories for subsequent analysis. Data were managed and analyzed using the R statistical software version 3.2.1 [19], and the Chi-square test for count data was used to assess independence in the contingency tables. The Bonferroni [20] correction was applied to decrease the probability of Type I error in multiple comparisons.

### Ethical approval

The protocol for this study deemed non-research (evaluation of a public health program) by the Institutional Review Boards of Emory University and the Centers for Disease Control and Prevention, the Ethics Committee of the Center for Health Studies (CES-UVG) and by the Vector Borne Diseases Program of the MSPAS. Written informed consent was obtained from all survey participants.

## Results

### The sample

Residents of 148 (88.1%) of the 168 total households in the four communities were interviewed. Among the 20 households not interviewed, 12 (7.1%) were excluded due to absence of a qualified adult respondent, 6 (3.5%) due to unwillingness to participate, and 2 (1.2%) due to less than 6 years in residence. There were no significant differences in household participation rates among the four villages.

The 148 interviews conducted in the four communities were as follows: 36 (24%) in Los Andes, 27 (18%) in Santa Isabel, 37 (25%) in Los Tarrales and 48 (33%) in La Estrellita. The self-reported age of participants ranged from 18 to 110, with a median age of 42, and 60% of respondents were female. There were no significant differences between communities in length of residence, which ranged from 6 to 110 years with a median of 36 years. The paired comparison format was presented orally to a subsample (108 or 73%) of interviewed households.

### 1) Illnesses affecting community residents

Rank order based on pairs analysis that reflected perceived seriousness of common illnesses, compared with results from the 1991 survey [12], are shown in Table 1. The 2014 post-elimination survey revealed that diabetes, blindness (*ceguera*), hepatitis, vomiting (*vomitos*) and diarrhea (*diarrea*) were the five illnesses considered most serious. This 2014 list contained only two (vomiting and dysentery) of the 5 priorities listed in the 1991 pre-MDA survey [intestinal worms (*lombrices*), measles (*sarampion*), vomiting, dysentery (*disenteria*), and pertussis (*tos ferina*)]. Intestinal worms dropped from first place in 1991 to sixth in 2014. Onchocerciasis (*filaria*) was ranked higher in the post elimination survey (eighth place) compared to the pre-MDA survey (13^th^ place). Diabetes, blindness and hepatitis, the illnesses considered most serious in the 2014 survey, did not appear among the top 15 conditions listed in 1991.

**Table 1:** Differences in perceived seriousness of onchocerciasis (as determined by “free-listing” and paired comparisons) in 2014 compared to 1991. Illnesses are listed in order of seriousness (greatest to least) as determined by paired comparison. The ‘(Rank)’ refers to the frequency the illness term was mentioned in the respondents’ free listings. Spanish terms are given in parentheses in italics. Dysentery (*disentería*), understood as painful diarrhea with blood, is different from diarrhea. URI (*resfriado*) = upper respiratory infection was not distinguished from influenza (*gripe*).

	2014 survey			1991 survey (Richards *et al*., 1991 [12])		
No.	Illness (*Spanish*)	Mention in free list (rank) N = 148	Count more serious N = 108	Illness (*Spanish*)	Mention in free list (rank) N = 145	Count more serious N = 40
1	diabetes (*diabetes*)	3 (11)	1191	intestinal worms (*lombrices*)	14 (12)	424
2	blindness (*ceguera*)	4 (9)	1171	measles (*sarampión*)	8 (15)	398
3	hepatitis (*hepatitis*)	3 (12)	1112	vomiting (*vómitos*)	33 (6)	376
4	vomiting (*vómitos*)	5 (8)	954	dysentery (*disentería*)	17 (10)	373
5	diarrhea (diarrea)	28 (6)	940	pertussis (*tos ferina*)	10 (14)	346
6	intestinal worms (*lombrices*)	4 (10)	864	diarrhea (*diarrea*)	75 (3)	342
7	fever (*fiebre*)	45 (2)	850	bronchitis (*bronquitis*)	4 (16)	331
8	onchocerciasis (*filaria*)	2 (15)	800	GI illness (*empacho*)	4 (16)	292
9	chickenpox (*varicela*)	3 (14)	673	fever (*fiebre*)	93 (1)	265
10	Stomach pain (*dolor de estómago*)	30 (5)	655	anemia (*anemia*)	10 (14)	264
11	cough (*tos*)	39 (3)	508	malaria (*malaria*)	19 (9)	213
12	headache (*dolor de cabeza*)	37 (4)	460	body/joint aches (*reumatismo*)	22 (8)	194
13	body pain (*dolor de cuerpo*)	27 (7)	403	onchocerciasis (*filaria*)	12 (13)	167
14	skin diseases (*granos*)	3 (13)	388	URI/influenza (*resfriado/gripe*)	55 (4)	152
15	URI/influenza (*resfriado/gripe*)	77 (1)	371	skin diseases (*granos*)	23 (7)	63

### 2) Onchocerciasis symptoms and transmission

**Fig 2:**
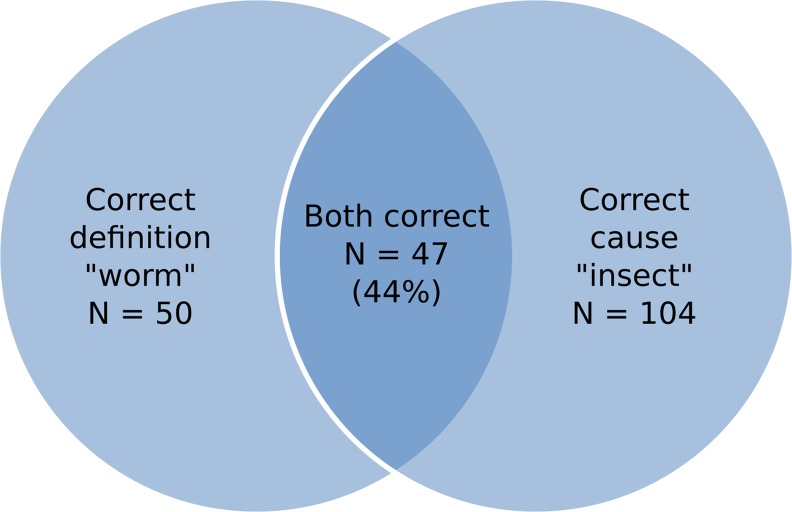
Venn diagram showing the survey participants’ knowledge of the correct definition and cause of onchocerciasis.

Ninety-four percent of respondents recognized the term *filaria* in the 2014 survey (compared to 100% in 1991). Most respondents (84%) were able to correctly describe the symptoms of onchocerciasis and almost all of these (97%) reported that the symptoms of *filaria* included the presence of nodules, cutaneous manifestations and eye disease. There was evidence that knowledge of onchocerciasis transmission and etiology had improved compared to 1991. Seventy percent knew that the disease was caused by the bite of an insect (compared to 50% in 1991) and 47% correctly defined the condition as being worm (compared to 39% in 1991). However, of the107 respondents in this survey who provided *either* a correct response for the definition (worm) *or* for the cause (insect bite) of the disease, only 44% knew both (Fig 2), which was not significantly different from the 35% who knew both in the 1991 survey (p = 0.19).

Ninety-three percent of all respondents reported knowing someone in the past in their community who had contracted the disease, and 73% reported having had onchocerciasis. Knowledge of having been personally infected was strongly related to age with older people more likely to have had the illness (p < .001, X^2^ = 37.67, df = 3). Of those previously infected, 63% recognized their own infection by the presence of subcutaneous nodules or visual impairment, while 37% reported that they had been diagnosed by uniformed brigade health workers of the MSPAS. Of the 34% who indicated that they had not had the disease, 89% of these had undergone an examination by palpation for nodules or a skin biopsy by brigade health workers.

### 3) The treatment of onchocerciasis

Ninety-nine percent of respondents confirmed that health workers had come to their communities to deliver pills (*las pastillas*) to treat onchocerciasis and of these, 99% reported having taken the medication. When asked if they knew the name of the tablets, 64% of those interviewed indicated they did and 96% of these correctly identified it as Mectizan or ivermectin. When asked how the tablet worked against *filaria*, 50% of the responses indicated the pill killed microfilaria, 36% said it killed *filaria* and 13% indicated that it prevented the disease (i.e., it was understood to be prophylactic). Seventy-six percent of the respondents knew that ivermectin could cause side effects (*molestias*), but no one could recall having seeing such adverse events after treatment for many years. When asked “Does the pill for *filaria* help to cure other illnesses?” 67 (45%) respondents indicated “yes”, of these 47 (70%) indicated that the pill cured intestinal worms, 15 (22%) said the pill cured ectoparasites such as lice (*piojos*) and scabies (*granos*) and 5 (8%) responded the pill cured both these conditions.

### 4) The reasons for halting MDA

The majority (69%) of respondents said that the MDA program had ended because the disease was no longer present in their communities, but almost a third (31%) did not know why MDA was halted. However, when all respondents were pressed by asking personal opinion, (“Do ***you*** believe that onchocerciasis has been eliminated in your community”?), over half (53%) of all respondents gave responses showing skepticism. Nineteen percent said they were unsure that elimination had taken place, while 34% were certain it had not (by responding ‘no’ to the question). When asked why they responded in this way, the ‘doubters’ said “because there are still people sick with la filaria” (72%) or that “there are still flies that transmit the disease” (28%). Several respondents also expressed concern about the children who had been too young to take the treatment, assuming that these untreated children were at greater risk than those who had benefited from the MDA program. There was a significant association between those who indicated that they missed receiving ivermectin treatment and those who expressed the opinion that that onchocerciasis may not have, or definitely had not, been eliminated in their community. (p < .05, X^2^ = 11.3, df = 1).

Ninety-three respondents (63%) said they missed receiving their twice per year ivermectin treatments; there were no age or sex differences associated with the desire to continue receiving the pill. When those who missed taking the pill were asked why, 87% answered that because the medicine was needed to prevent onchocerciasis, with the remaining 13% wanting to take treatment to treat their intestinal worms (*lombrices*) and/or ectoparasites (*piojos*, *granos*).

### 5) The post-treatment period

Educational and promotional activities during the 3-year PTS period (2012–2014) took three forms. The first was the MSPAS house to house ‘briefings’ at the individual community level that involved the same uniformed brigades that had provided MDA and conducted the monitoring and evaluation activities associated with the program. The second form of educational activity involved musical community celebrations, crafting activities, and parades (‘victory marches’) through the streets of larger communities and towns in the CEZ. These celebratory events involved a non-profit artistic troupe called “Caja Lúdica” (http://www.cajaludica.org/). The artists and the MSPAS health workers dressed in colorful costumes as the black flies, worms (*filaria*), and Mectizan bottles; the artists often performed acrobatics while on stilts. Residents of the communities were encouraged to participate in the parades and associated activities. The third approach was to provide information through short radio ‘jingles’ on onchocerciasis. A critical element in all forms of community health education messaging was to ask the population to keep alert to new cases of onchocerciasis (especially the appearance of nodules).

Forty three percent interviewees reported that they had been exposed to one or both of these PTS educational and promotional activities. Most of these (54%) had attended at least one community briefing with a health worker, with 33% having attended a large Caja Lúdica celebration. The remainder (13%) reported having heard about the MSPAS visits or large celebrations from a neighbor. Only 6% of respondents reported that they had attended a briefing, or participated in a celebratory parade, in a community other than their own. Among persons interviewed in La Estrellita, where Caja Lúdica celebration held a parade, the overwhelming majority (99%) preferred the large celebrations to MSPAS briefings, saying that the former were more entertaining and informative. Only one person reported hearing a radio jingle related to onchocerciasis elimination.

When asked “If someone in your family presented symptoms of *filaria* what would you do?”, 89% responded that they would go to the health clinic. However, when asked “How quickly would you go to the health clinic?”, only 56% replied that they would do so immediately.

With the cessation of the six monthly MDA program, Ministry of Health community outreach activities were perceived to have decreased. Although 117 (79%) of the respondents indicated that the community had received visits from health workers, only 39 (28%) identified these visits as related to onchocerciasis. The most frequent health brigade outreach activities, identified by 72% of respondents, were related to vector control for malaria and dengue, or childhood vaccination campaigns.

## Discussion

This is the third in a series of studies conducted to investigate evolving community knowledge, attitudes and practices (KAP) related to the ivermectin mass drug administration (MDA) program to eliminate onchocerciasis from Guatemala. Many of the questions and methods used in this report were replicates of previous instruments, and were applied in and around the same communities, in order to get a longitudinal view point of KAP evolution. We were also interested in exploring new lines of questioning designed to understand the KAP pertaining to the withdrawal of ivermectin from communities after their two decade experience with the drug. Implications for ongoing surveillance and health education activities during the post-endemic period were identified. Our approach may be useful in other neglected tropical disease MDA programs where treatment is being stopped (such as lymphatic filariasis and trachoma).

Our first KAP survey (published in 1991) was conducted in communities that had not yet experienced ivermectin but where ministry of health (MSPAS) outreach nodulectomy surgical services had been offered for decades [16]. The top five diseases provided in a free listing paired analysis at that time were intestinal worms, measles, vomiting, dysentery, and pertussis. Two of top five terms (vomiting and diarrhea) again were found in the 2014 priorities, although many respondents differentiate dystentery from diarrhea based on the severity of symptoms (dystenery being much more severe). In 2014 there were three new terms (diabetes, blindness, and hepatitis) in the top five diseases. Intestinal worms dropped from first to sixth place between 1991 and 2014, perhaps due to a perceived lower prevalence resulting from years of ivermectin treatment (effective against the human roundworm *Ascaris lumbricoides)*. This conclusion is supported by the finding that 35% of respondents said that ivermectin was effective against intestinal worms.

Both the Pre-MDA 1991 and the Post-elimination 2014 surveys showed onchocerciasis was not perceived as a particularly serious illness, and in both cases less serious than intestinal worms. In the present survey, however, a greater importance of (the now eliminated) onchocerciasis resulted in its move up to 8^th^ place (from 13^th^ in 1991). This is likely a result of the health promotion campaign that was designed to increase knowledge of *filaria* and its consequences. Perhaps the same information campaign resulted in blindness being listed as the 2nd most serious disease afflicting the communities, in spite of the fact that blindness is a rare occurrence in these communities.

Almost all of the respondents either had had onchocerciasis or a diagnostic evaluation for onchocerciasis. Older individuals were more likely to have had the infection or have known someone who had had *filaria*. The frequent experience with diagnostic evaluations undoubtedly resulted from the fact that these villages were sentinel areas for the MDA program and the inhabitants were subject to nodule palpation and skin snipping tests every four years. Given this experience with the disease and the program, it was disappointing that less than half (44%) of respondents knew the basic fact that onchocerciasis was caused by a worm transmitted by an insect. This was statistically no improvement over the 39% who knew this fact in 1991. Almost all respondents reported having consumed ivermectin, and there were no negative opinions voiced about *la pastilla*.

Although the majority (69%) of respondents said that the MDA program had ended because the disease had been eliminated from their communities, when participants were asked their personal opinion, half of all respondents were skeptical that elimination had really occurred. These ‘doubters’ said the basis for their belief was that there were still people ill with onchocerciasis, or that the disease vectors were still there transmitting the disease. Some community members were concerned that those children who had been too young to take the treatment were now at more risk for coming down with onchocerciasis compared to older persons who had benefited from the MDA program. The doubters were significantly more likely to say they wanted the MDA reinstated for these reasons.

Onchocerciasis elimination guidelines call for national programs to assure through health education activities that all treated communities be given clear understanding of reasons behind a decision to suspend ivermectin MDA. Of particular importance to this process is the maintenance of the lines of communication between the health team, community leaders, former MDA distribution volunteers, and the communities during the three year post treatment surveillance (PTS) period [21]. In Guatemala, various activities were undertaken to keep community interest alive with respect to the declaring of elimination and the stopping of MDA, as well as the need for the community to maintain vigilance in case of recrudescence. Three methods (MSPAS outreach, Caja Lúdica celebratory events in selected communities, and radio jingles) were utilized to accomplish these health educational goals in formerly endemic communities in the CEZ.

Our survey found that just 35% of interviewees had been reached directly by one or more of these PTS educational and promotional activities. Most participants got the PTS message in their villages from MSPAS outreach, and in our study only residents in La Estrellita were exposed to a Caja Lúdica event. Only one person reported having heard a radio message related to onchocerciasis. On the other hand, evidence of the willingness of communities to participate in the surveillance process was found in the 89% of respondents who indicated they would go to a clinic or MSPAS worker if they (or a family member) developed *filaria*. Given the high level of symptom recognition of the disease, this suggests that passive surveillance can be relied upon for reporting the appearance onchocerciasis symptoms, assuming the MSPAS is sufficiently organized and prepared to receive and evaluate this potential source of information. Of concern was the finding that residents perceived that overall MSPAS community outreach presence in their communities had decreased since the end of the MDA program.

Sixty three percent of respondents said they missed receiving their twice per year ivermectin treatments, 87% of whom wanted to continue to take the tablets to prevent onchocerciasis, and the remaining 13% to treat their intestinal worms and/or ectoparasites. This is consistent with a considerable appreciation of the key and visible ancillary benefits of ivermectin aside from treatment for *filaria*. These findings suggest the likelihood that the communities will miss the ivermectin campaign more and more over time, especially since general deworming programs (which employ albendazole or mebendazole) do not have the same efficacy as ivermectin does against scabies and lice, and MDA for intestinal parasites targets school age children, not the entire population [22].

Our second KAP survey (published in 1995) was undertaken to better understand the considerable rejection of the ivermectin MDA program. In Santa Isabel in particular, fewer than 60% of the treatment eligible residents accepted ivermectin treatment when offered during the initial three treatment cycles [13]. All participants (and especially those in Santa Isabel) feared the adverse reactions (*molestias*) that followed ingestion of the tablets. It is therefore notable in the 2014 survey that over 95% of respondents reported having consumed ivermectin, and there were no negative opinions voiced about the medicine. In other words, unlike in 1995, we found no variations between the four communities surveyed (which included the once recalcitrant Santa Isabel) in acceptance of the medicine, or in the opinion that MDA ought to be reinstated. The fact that there were no concerns about adverse events in recent years attests to the temporary nature of adverse events associated with MDA, and the evolution of a prevailing belief in the safety of ivermectin.

There were several limitations to this study. The most important was that the interviews took place in four of the ten sentinel communities used to monitor the impact of the MDA program. As such, these populations had much more contact with MSPAS brigades than other communities in the Central Endemic Zone, and our results are likely biased toward a greater knowledge and importance of onchocerciasis. The respondents’ answers related to the recent experiences with diagnostic activities (such as skin snipping and nodule palpation) resulted from a sentinel community experience. MSPAS personnel would also have engaged some local residents to assist them in catching the vectors; one might expect that that would have led to a greater knowledge that *filaria* was transmitted by an insect. Second, previous KAP surveys were conducted in these same communities and some of the respondents may have participated in these earlier surveys. If there was previous participation it might have influenced answers to questions. However, this was unavoidable given that our design sought to return to areas previously studied in order to get a longitudinal view point of KAP evolution. Lastly, we noted one third of the respondents were skeptical that elimination had really occurred, and most of these said the basis for their belief was that there were still people ill with onchocerciasis. Unfortunately, we failed to explore that response by asking how many people they knew at that very moment who had *filaria* in the community, how they knew that those neighbors had *filaria* (e.g., what were their symptoms), and why those individuals had not sought clinical or MSPAS brigade services. That additional information would have been very informative in judging how potential passive community surveillance for onchocerciasis could be better tailored for improved specificity of reports being provided within the contextual milieu.

### Conclusion

The purpose of the present study was to evaluate KAP towards onchocerciasis and ivermectin among residents in the post endemic CEZ. A major interest in this study was to determine what community residents thought about the end of the ivermectin MDA program. We found that three years after cessation of a two decade long ivermectin MDA program for onchocerciasis elimination in Guatemala, many residents wanted the ivermectin MDA program reinstated because of their uncertainty about onchocerciasis elimination, and their desire to have treatment for intestinal worms and ectoparasites. The vast majority of those questioned said that they would seek medical attention immediately if a family member presented symptoms of onchocerciasis (especially the finding of a nodule), which is a practice very important for ongoing post endemic surveillance. The ministry of health outreach services, which has been reduced since the halting of the ivermectin MDA program, should be properly resourced to be prepare to address such reports, and seek to understand ongoing concerns of the populations residing in onchocerciasis post MDA Guatemala.
